# The impacts of total quality management practices in Algerian higher education institutions

**DOI:** 10.3389/fpsyg.2022.874209

**Published:** 2022-08-25

**Authors:** Fethia Yahiaoui, Khalil Chergui, Nesreddine Aissaoui, Said Khalfa Mokhtar Brika, Imane Ahmed Lamari, Adam Ahmed Musa, Mohmmad Almezher

**Affiliations:** ^1^University of Oum El Bouaghi, Oum El Bouaghi, Algeria; ^2^University of Bisha, Bisha, Saudi Arabia; ^3^Department of Management Administration, Faculty of Economics, White Nile University, Kosti, Sudan

**Keywords:** quality, quality of higher education, TQM, mixed-method approach, Algerian universities

## Abstract

Algerian universities rely on total quality management. TQM is one of the most successful strategic options for improving the quality of higher education. In addition, achieving academic accreditation and progress in international rankings. The study aims to address relevant contemporary issues by examining the impact of total quality management on the quality of higher education. The data were analyzed using a mixed-method approach; the study was done as a survey, with data collected *via* questionnaires issued to 610 students. The questionnaire included Likert scale items that were quantitatively evaluated and modeled using structural equation modeling (SEM) using Amos to accomplish the path analysis of the research model. Furthermore, qualitative data were acquired through interviews with 24 professors who are members of the Quality Cells, and qualitative data were evaluated using content analysis with NVivo. The study findings revealed that TQM has a direct and significant impact on the quality of graduates, scientific research, and community service in Algerian universities. The main results have been presented, and recommendations for future research are made.

## Introduction

Since “quality gurus” such as Walter Shewhart, Edwards Deming, Joseph Juran, Philip Crosby, Armand Feigenbaum, Kaoru Ishikawa, and others underlined the critical role of Total Quality Management and its importance in obtaining various organizational benefits. Several researchers have expressed an interest in writing about it and its consequences. Several researchers consider TQM as a source for improving performance (Pike and Barnes, 1995; Reed et al., 1996; Kumar et al., 2009; García-Bernal and Ramírez-Alesón, 2015; Anil and Satish, 2019). TQM is viewed as a source of competitive advantage (Powell, 1995; Reed et al., 2000; Douglas and Judge, 2001; El Shenawy et al., 2007; Ferdousi et al., 2018). It is also regarded as a means of enhancing financial performance and achieving profitability (Hansson and Eriksson, 2002; Selladurai, 2002; Wayhan and Balderson, 2007; Milovanovic, 2014). It also served as a source of customer satisfaction (Forza and Filippini, 1998; Sit et al., 2009; Ooi et al., 2011; Sheikholeslam and Emamian, 2016). Many researchers see it as a means of achieving social responsibility (Ghobadian et al., 2007; Aguilar and Bernardo, 2013; Benavides-Velasco et al., 2014; Jalilvand et al., 2018; Abbas, 2020).

On this basis, research into total quality management practices and their applications in various fields and sectors began, including TQM in small and medium enterprises (Ghobadian and Gallear, 1996; Adriana Tisca et al., 2015; Hilman et al., 2020), TQM in high-technology companies (Price and Chen, 1993; Yang, 2006), and Total quality management in service institutions, such as hotels, hospitals, schools, public administrations … etc. (Milakovich, 1991; Martin, 1993; Bradley, 1994; Short, 1995; Sureshchandar et al., 2001; Bon and Mustafa, 2013).

For several years great effort has been devoted to the study of TQM and its core values, tools, methods, practices, and impacts in the educational sector (Vlašić et al., 2009; Sallis, 2014; Mukhopadhyay, 2020). Including investigations that focused on TQM in higher education, this problem has been researched in several ways. First, TQM practices in universities and higher education institutions (Abdulameer et al., 2014; Ahmed and Ali, 2016), second, TQM implementation in higher education (Taylor and Hill, 1992; Anderson, 1995; Sirvanci, 2004; Meirovich and Romar, 2006; Venkatraman, 2007), and third, TQM benefits, results, and impacts in higher education (Shahdadnejad and Alroaia, 2013; Aminbeidokhti et al., 2016; Psomas and Antony, 2017; Ullah et al., 2018; Padró et al., 2020).

Based on how the quality approach was applied by various quality gurus to this particular context of research, Walter Shewhart began to focus on controlling processes, using a statistical method to make quality relevant not only for the end product (graduate students) but also for the teaching and learning process that produced them (Shewhart and Deming, 1986). As for Edwards Deming, one of the well-known tools that proved effective in improving the quality of services provided by higher education institutions was the PDCA cycle (plan, do, check, and act; Deming, 1991). According to Joseph Juran, the use of the Juran Trilogy (quality planning, quality control, and quality improvement) has been shown beneficial in various contexts to evaluate and improve the quality of services delivered in higher education (Juran, 2005; Sok and Taib, 2012). Philip Crosby’s contributions were notable for 14 quality management points and the concept of zero defects to achieve high-quality levels in higher education institutions (Crosby, 2005; Farooq et al., 2007). Armand Feigenbaum proposed Total quality control as a system for integrating the quality development, quality maintenance, and quality improvement efforts of various groups in higher education institutions to control quality costs (prevention costs, appraisal costs, internal failure costs, and external failure costs) and provide complete customer satisfaction (Feigenbaum, 1991). Ishikawa is well-known for proposing several practical tools for quality improvement, such as the Ishikawa fishbone diagram, which aims to improve quality by identifying the primary and secondary causes of a specific quality problem (Ishikawa and Loftus, 1990).

In Algerian higher education institutions, several academics have studied TQM (Khelifa et al., 2013; Barka and Ilhem, 2016; Keffane et al., 2020; Belimane and Chahed, 2021). They focused on implementing TQM principles in Algerian higher education while ignoring TQM’s impact at various levels. This is what constitutes a research gap for previous research that must be addressed by the study, as the various effects of applying total quality management principles in higher education must be identified and measured to improve the quality of students and graduates, the quality of scientific research, the quality of community services, and so on. Detecting the effects of the TQM application using mixed methods (qualitative and quantitative approaches).

Accordingly, the problem of the study is to reveal the impacts of applying Total Quality Management practices in Algerian higher education institutions to improve the quality on three levels: quality of graduates, quality of scientific research, and quality of community service. Using mixed methods from the customer’s or student’s perspective is advantageous (quantitative and qualitative).

### Theoretical background

In higher education, Total Quality Management is defined as a management philosophy, a management method, an integrated system, a continuous improvement approach, and a change approach for achieving excellence. This is what Hansson and Klefsjö (2003) referred to as an integrated system, which involves the adoption of a set of core values, techniques, and tools, where they believed that TQM’s core values were the foundation for their application’s success. Theoretical literature refers to these core values as principles (Morrow, 1997; Helms et al., 2001; Mar Fuentes-Fuentes et al., 2004; Aguilar and Bernardo, 2013). It is also referred to as a series of practices (Ooi et al., 2011; Zwain et al., 2014; Ahmed and Ali, 2016; Khan et al., 2019). However, most researchers agree on their nature and number, as shown in Table 1.

**Table 1:** TQM practices in higher education.

Practices	Ünal, 2001	Sabihaini et al., 2010	Kysilka and Medinschi, 2011	Zakuan et al., 2012	Abdulameer et al., 2014	Ahmed and Ali, 2016	Padró et al., 2020
Customer focus	√	√	√	√	√	√	√
Management commitment	√	√	√	√	√	√	√
Total employee involvement	√	√	√	√	√	√	√
Process approach	–	–	√	–	√	√	–
A systematic approach to management	–	–	√	–	√	√	–
Continuous improvement	√	–	√	√	√	√	√
Fact-based decision-making	√	√	√	√	√	√	√
Mutually beneficial supplier relations	–	–	√	–	√	√	√

TQM, as well as the eight practices listed in Table 1, interact with internal and external quality assurance as to the foundation and process of attaining this, allowing the quality of higher education to be achieved (Bogue, 1998; Welsh and Dey, 2002; Moldovan, 2012; Asif and Raouf, 2013; Tight, 2020). In higher education, there are five major approaches to quality that can be identified: exceptional, perfection, fitness for purpose, value for money, and transformative (Harvey et al., 1993; Harvey and Williams, 2010). Higher education quality, according to Robert Birnbaum, is a multidimensional notion with three basic dimensions: the individual dimension (higher education contributes the formation of human competencies through the educational service directed to students), the eligibility dimension (higher education institutions must meet educational and international standards based on academic experts’ assessments), and the social dimension (degree of satisfying the needs of the various actors in the society; Papadimitriou, 2011).

Quality of higher education is related to the extent to which university activities (academic, administrative, and community) achieve their goals and meet the quality standards that are expected of them, as measured by a set of pre-defined characteristics known as dimensions, indicators, and quality standards. These dimensions are primarily represented in Table 2.

**Table 2:** Quality dimensions in higher education.

Dimensions	Cave, 1997	Patrick and Stanley, 1998	Kwan and Ng, 1999	Gibbs, 2010	Ullah et al., 2011	Florida and Quinto, 2015	Yeung, 2018
Quality of graduates	√	√	√	√	√	√	√
Quality of scientific research	√	√	√	√	√	√	√
Quality of community service	√	–	√	√	–	√	√

Total quality management is viewed in the theoretical literature as an integrated set of dimensions that are applied as a single system and are not isolated from one another (Hansson and Klefsjö, 2003). As for the quality of higher education, each dimension is measured separately, and the partial correlations between the study variables might be imagined as follows.

### TQM and quality of graduates

Many trends have suggested that TQM has an impact on increasing the quality of students from higher education institutions. According to many researchers, TQM is thought to have direct effects on improving graduate quality and indirect effects through improving educational process quality (Dahlgaard et al., 1995; De Jager and Nieuwenhuis, 2005; Dahil and Karabulut, 2013).

Willis and Taylor (1999), for one, emphasized that total quality management is built on focusing on the customer and studying his needs, which are then converted into professional standards of employers that enable graduates to work and achieve excellence in a highly competitive business environment. Sakthivel et al. (2005) found that the five TQM components (Top management commitment, Course delivery, Campus facilities, Courtesy, and Customer feedback and improvement) have an impact on the quality of educational service and students’ academic performance satisfaction. According to Dahil and Karabulut (2013), TQM is a process that develops the educational system and the training of qualified personnel who can match the public’s expectations. In his studies in Spanish universities using a graduate employability survey, Martínez-Gómez et al. emphasized that TQM training and knowledge help graduates to have acquired the competence needed to perform their job successfully (Martínez-Gómez et al., 2018, 2020). Furthermore, Abbas et al. (2021), highlighted QMS as a key component in improving students’ employability and industry acceptance. In Algerian universities (Khelifa et al., 2013; Keffane et al., 2020) emphasized the existence of several effects of total quality management, including improving the quality of graduates.

*H1*: There is a direct and significant impact of TQM on the Quality of graduates in Algerian universities.

### TQM and quality of scientific research

According to Hemlin (1993), scientific quality is measured by quality indicators (citations, awards, and peer review), the scientist’s research effort, the researcher himself/herself (knowledge, skills, and abilities), the research environment, the scientific effect, an institution’s research policy and organization, and finally financing. Anninos (2007) indicated that high-quality scientific research is easier than achieving high-quality instruction since the quantitative and qualitative indicators are unambiguous, and quality management systems aid in reaching research excellence. Previous scholars affirmed that the quality of scientific research is impacted in a variety of different forms by total quality management (direct and indirect; Black, 1995; Kanji et al., 1999; Sallis, 2014).

For his part, Jusoh et al. (2008) stated that the use of TQM components is critical in higher education, research and development, and technology transfer, particularly because it is the foundation for the Malaysian economy’s development. Some believe that whole quality management adds to academic success, including both teaching and scientific research (Mashagba, 2014; Zwain et al., 2017). In addition, Lanati (2019) mentioned in detail in his book “Quality Management in Scientific Research—Challenging Irreproducibility of Scientific” quality tools for the scientific research (Pareto Chart, Ishikawa, Control Charts, Flow Chart, Decision-Making and Problem Solving Tools, Knowledge Management, and Team Work) and quality methods for the scientific research (Project Management, Failure Mode and Effect Analysis, Design of Experiment, Lean Management and Six Sigma). In Algerian universities, Khelifa et al. (2013), Barka and Ilhem (2016), Keffane et al. (2020) and Belimane and Chahed (2021)pointed out that there are different effects of total quality management in higher education institutions, including improving the quality and output of scientific research.

*H2*: There is a direct and significant impact of TQM on the Quality of scientific research in Algerian universities.

### TQM and quality of community service

Many studies have established that total quality management provides a role in achieving social responsibility and that there is an integrative relationship between the two in various industries (Ghobadian et al., 2007; Aguilar and Bernardo, 2013; Benavides-Velasco et al., 2014; Jalilvand et al., 2018; Abbas, 2020). In the case of higher education, there is a potential impact on achieving high-quality community services as a social responsibility factor. Many researchers emphasized that total quality management has many effects in higher education institutions, allowing it to influence society and achieve its societal commitments, what is known as quality of community service (Barnett, 1992; Couch, 1997; Kanji et al., 1999; Koch, 2003; Sahney et al., 2004; Sallis, 2014). According to Khoja et al. (2017), the assurance of the quality of community services in Libyan higher education and emerging countries is related to the necessity to connect the aspects of overall quality management with the dimensions of completion in the sustainable quality management (SQM) model. Yeung (2018) identified three levels of sustainable development in higher education: organizational stakeholder participation, educational goals, and community need realization; teacher awareness of environmentally friendly issues, competency-based concepts, and providing real-world exposure to learners; and learner role definition. To achieve these levels, he emphasized the importance of facilitating effective integration of the ISO 9001 quality management system (QMS), ISO 26000 guidelines for corporate social responsibility (CSR), and program accreditation requirements. Banerjea (2018) emphasized that total quality management has a strong ethical dimension, calling for the importance of taking into account the interests of stakeholders, as it is considered a foundation and a catalyst for effective corporate social responsibility in the higher education sector in his study to explore the importance of TQM and CSR to promote education and various corporate initiatives in community. Castillo (2020) reaffirmed the necessity to develop and implement TQM concepts to all aspects of higher education academic units, including teaching, research, community services, and administrative support. Furthermore, Nogueira emphasized in his different studies that TQM and CSR are two increasingly significant dimensions in creating a sustainable and hopeful future for all stakeholders, and that they are important dimensions in achieving a sustainable and promising future for all stakeholders (among others, students and workers employers, and society in general; Nogueiro et al., 2011, 2017, 2022). In Algerian universities, Khelifa et al. (2013) and Keffane et al. (2020) emphasized the existence of numerous effects of total quality management, including improving the quality of community service.

*H3*: There is a direct and significant impact of TQM on the Quality of community service in Algerian universities.

The study’s research model might be depicted as follows, based on the links between total quality management and higher education quality.

## Materials and methods

### Data collection

#### Quantitative data and sample selection

The purpose of this study was to determine the impact of TQM on higher education quality in Algerian universities. The research was carried out as a survey, with data collected using a standardized questionnaire delivered to students at all levels. According to the Yamane (1967) formula, the first sample consisted of 610 students: *n* = *N*/1+ Nd2 = 1,777,304/1 + (1,777,304*0.052) = 400 (Adam, 2020). Because of the projected difficulty in getting lists of all Algerian students, 610 questionnaires were gathered using a snowball sample (non-probability sample) related to network sampling (Handcock and Gile, 2011).

The questionnaire included constructs that would be tested to do quantitative analysis. The following is a five-point Likert scale that was used to express construct measurement items: 1 means severely disagree, 2 means disagree, 3 means medium agree, 4 means agree, and 5 means strongly agree. In addition to demographic data, the questionnaire included two major constructs: TQM (continuous improvement, customer focus, fact-based decision-making, management commitment, mutually beneficial supplier relationships, process approach, systematic approach to management) with eight dimensions, and quality of higher education with three dimensions (quality of graduates, quality of scientific research, and quality of community service).

Cronbach’s alpha and Guttman split-half were used to calculate reliability and validity, which was done using SPSS software (version 25). The validity and reliability coefficients of the questionnaire constructs are shown in Table 3.

**Table 3:** Validity and reliability statistics.

Constructs	Cronbach’s alpha	Guttman split-half	N of items	Number of cases
Customer focus	0.953	0.933	4	610
Management commitment	0.961	0.985	4	610
Total employee involvement	0.941	0.945	4	610
Process approach	0.965	0.945	4	610
Systematic approach to management	0.971	0.970	4	610
Continuous improvement	0.963	0.950	4	610
Fact-based decision-making	0.957	0.941	4	610
Mutually beneficial supplier relations	0.943	0.949	4	610
TQM	0.994	0.990	32	610
Quality of graduates	0.977	0.989	10	610
Quality of scientific research	0.977	0.975	10	610
Quality of community service	0.991	0.993	10	610
Quality of higher education	0.992	0.965	30	610

Table 3 provides the summary statistics for Validity and Reliability; it shows that the reliability coefficients (Cronbach’s Alpha) are 0.994 for TQM and 0.992 for quality of higher education, which are within the acceptable limit as per Bland and Altman (1997). It presents also that the Validity coefficients (Guttman split-half) are 0.990 for TQM and 0.965 for quality of higher education, which are within the allowed range according to Jackson (1979). This indicates that the questionnaire of this study is suitable for conducting research and drawing conclusions.

#### Qualitative data

In management sciences, the interview is considered an effective way to collect qualitative data, and in our study, it was used by organizing it with professors who specialize in the subject, and 24 professors are members of the Quality Cells, which monitor issues of higher education quality and quality management in Algerian universities. This is done to find a study model test from the perspective of specialized academics, not merely students.

### Methods and analysis approaches

#### Quantitative methods

We have used structural equation modeling (SEM) through IBM SPSS Amos 25 to assess the relationships in the research framework and test the hypothesis. Nachtigall et al. (2003) indicate that the comparison of the model to empirical data is the main feature of SEM. This comparison generates so-called fit statistics, which evaluate the model’s fit with the data. Using this method or what is known as covariance-based structural equation modeling (CB-SEM) requires three conditions (Lowry and Gaskin, 2014). Suitable for confirmatory studies and the model must be precisely delimited between the variables, appropriate for large samples (the sample in this study was 610), requires a normal distribution of the data shown in Table 4.

**Table 4:** Tests of normality.

Constructs	Skewness	Kurtosis
Customer focus	1.508	1.48
Management commitment	1.094	0.047
Total employee involvement	1.547	1.624
Process approach	1.24	0.819
A systematic approach to management	1.357	1.032
Continuous improvement	1.34	1.057
Fact-based decision-making	1.296	0.918
Mutually beneficial supplier relations	1.196	0.58
TQM	1.341	1.084
Quality of graduates	0.968	−0.106
Quality of scientific research	1.232	0.671
Quality of community service	0.739	−0.852
Quality of higher education	0.946	−0.143

A significant divergence from normality, according to West et al. (1995), is defined as an absolute skewness value > 2 and an absolute kurtosis (proper) value > 7. Table 4 shows that all of the research variables’ absolute values are less than 2 for skewness and less than 7 for kurtosis, indicating that the data follow a normal distribution.

#### Qualitative methods

The use of qualitative methods in addition to quantitative methods, referred to as the mixed method, is due to the importance of the qualitative approach in compensating for the shortcomings of quantitative methods. Whereas these methods rely primarily on analyzing the opinions of specialists (in our study, they were represented by 24 professors who are members of the Quality Cell) who answer the subject in an accurate and extensive manner (*via* the open questions of the interview guide), allowing a good understanding of the topic and confirmation of the quantitative analysis results, and this emphasizes the importance and benefits of qualitative methods using NVivo (Madey, 1982; Henwood and Pidgeon, 1992; Edwards-Jones, 2014; Almalki, 2016; Jackson and Bazeley, 2019).

NVivo is a software program that can be used to save, manage, and analyze qualitative data and open-ended questions (Edwards-Jones, 2014). Visualization techniques (thematic analysis, cluster analysis and cognitive mapping were used to link two variables: TQM and quality of higher education, to confirm the study model qualitatively, and test the degree of its agreement) and thought experiments can also help to clarify what might be useful questions (Jackson and Bazeley, 2019).

## Results

The statistics and SEM findings are presented in this section to ensure hypothesis testing and the study model.

### Descriptive statistics

Table 5 provides a summary of the descriptive statistics for the study sample.

**Table 5:** Descriptive statistics of the study sample.

Variables	Categories	Frequency	Percent	Valid percent	Cumulative percent
Gender	Male	387	63.4	63.4	63.4
	Female	223	36.6	36.6	100.0
Age	Less than 30 years	222	36.4	36.4	36.4
	30–40 years	211	34.6	34.6	71.0
	40–50 years	119	19.5	19.5	90.5
	More than 50 years	58	9.5	9.5	100.0
Edu. Level	Bachelor	224	36.7	36.7	36.7
	Master	200	32.8	32.8	69.5
	Doctorate	186	30.5	30.5	100.0
Specialty	Economic sciences	198	32.5	32.5	32.5
	Social and human sciences	112	18.4	18.4	50.8
	Law and political sciences	84	13.8	13.8	64.6
	Literature and languages	116	19.0	19.0	83.6
	others	100	16.4	16.4	100.0
	Total	610	100.0	100.0	

Table 5 summarizes the demographic characteristics of the study sample, revealing that the majority of respondents are male (63.4%), less than 30 years (36.5%), and most of them are undergraduate students (36.7% for bachelor’s degree and 32.8% for master degree), the study in different disciplines, the most important are Economic sciences (32.5%) and Literature and languages (19%). This explains a large number of undergraduate students in Algerian universities compared to postgraduate students, as well as the nature of the study sample (Bouchikhi and Zine, 2017). These characteristics, in turn, affect the students’ answers regarding the study variables presented in Table 6.

**Table 6:** Descriptive statistics of study variables.

Study variables	Minimum	Maximum	Mean	Std. deviation
Customer focus	1.00	5.50	1.8561	1.18015
Management commitment	1.00	5.00	1.9877	1.28096
Total employee involvement	1.00	5.50	1.8639	1.15840
Process approach	1.00	5.00	1.9398	1.19427
Systematic approach to management	1.00	5.00	1.9135	1.19951
Continuous improvement	1.00	5.00	1.9066	1.18014
Fact-based decision-making	1.00	5.00	1.9266	1.20244
Mutually beneficial supplier relations	1.00	5.00	1.9791	1.21256
TQM	1.00	5.13	1.9217	1.17680
Quality of graduates	1.00	5.00	2.0546	1.26201
Quality of scientific research	1.00	5.00	1.9172	1.18375
Quality of community service	1.00	5.00	2.1638	1.40244
Quality of higher education	1.00	5.00	2.0452	1.25184

Table 6 shows the descriptive statistics for the study variables. The majority of respondents chose the option of weak commitment to apply all TQM principles (mean = 1.9217), as well as the option of weak attainment of higher education quality standards (mean = 2.0452), as indicated by the small dispersion of the two variables based on the standard deviation. This explains several things, including Algerian universities’ tardiness in implementing total quality management principles and their commitment to internal quality assurance and self-evaluation processes, as well as their ranking in the international classification of universities for higher education quality (El Hassan, 2013; Guessoum, 2019).

### Correlation matrix

Table 7 shows the correlation matrix of study variables and constructs.

**Table 7:** Correlation matrix.

Variables and constructs		Customer focus	Management commitment	Total employee involvement	Process approach	Systematic approach management	Continuous improvement	Fact-based decision-making	Mutually beneficial supplier relations	TQM
Quality of graduates	R	0.896^**^	0.881^**^	0.928^**^	0.910^**^	0.888^**^	0.907^**^	0.891^**^	0.913^**^	0.920^**^
	Sig.	0.000	0.000	0.000	0.000	0.000	0.000	0.000	0.000	0.000
Quality of scientific research	R	0.950^**^	0.926^**^	0.944^**^	0.968^**^	0.964^**^	0.973^**^	0.961^**^	0.970^**^	0.977^**^
	Sig.	0.000	0.000	0.000	0.000	0.000	0.000	0.000	0.000	0.000
Quality of community service	R	0.809^**^	0.785^**^	0.808^**^	0.861^**^	0.864^**^	0.862^**^	0.857^**^	0.877^**^	0.858^**^
	Sig.	0.000	0.000	0.000	0.000	0.000	0.000	0.000	0.000	0.000
Quality higher education	R	0.903^**^	0.881^**^	0.911^**^	0.932^**^	0.925^**^	0.934^**^	0.922^**^	0.940^**^	0.937^**^
	Sig.	0.000	0.000	0.000	0.000	0.000	0.000	0.000	0.000	0.000

** Correlation is significant at the 0.01 level (2-tailed).

Table 7 shows that both TQM principles and Quality of Higher Education constructs have a significant positive correlation, with all correlation coefficients being strong (greater than 0.8 and significant at the 0.01 level). This is explained by three major factors: first, the respondents’ answers were similar in terms of total quality management principles and higher education quality; second, the positive impact of quality assurance and self-assessment efforts in Algerian universities on higher education quality; and third, despite the delay in implementing total quality management and committing to quality standards, Algerian universities are committed to quality (El Hassan, 2013; Badran et al., 2019; Guessoum, 2019).

### SEM results

**Figure 1:**
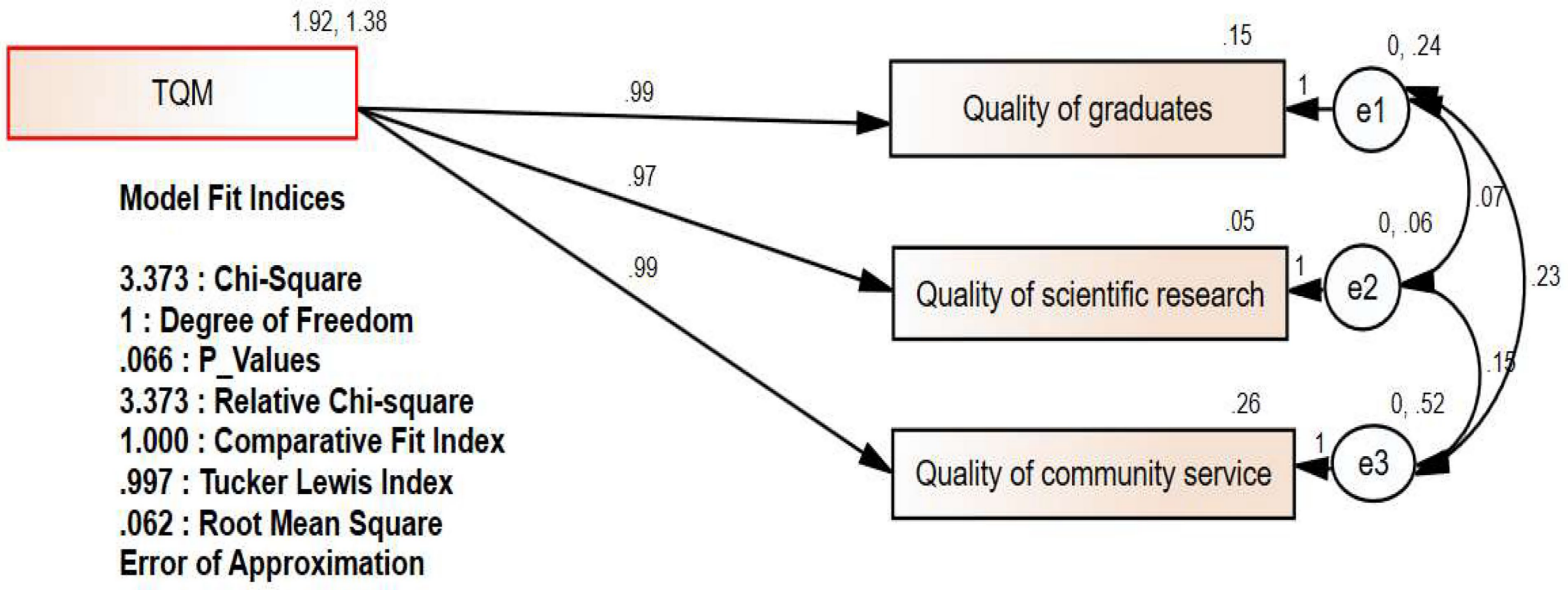
Path analysis model.

The path analysis model for confirmatory factor analysis was used to assess the study’s hypotheses and model, as it is considered an effective technique for doing so, as stated by Stage et al. (2004). The path analysis model’s outputs are shown in Figure 1.

**Figure 2:**
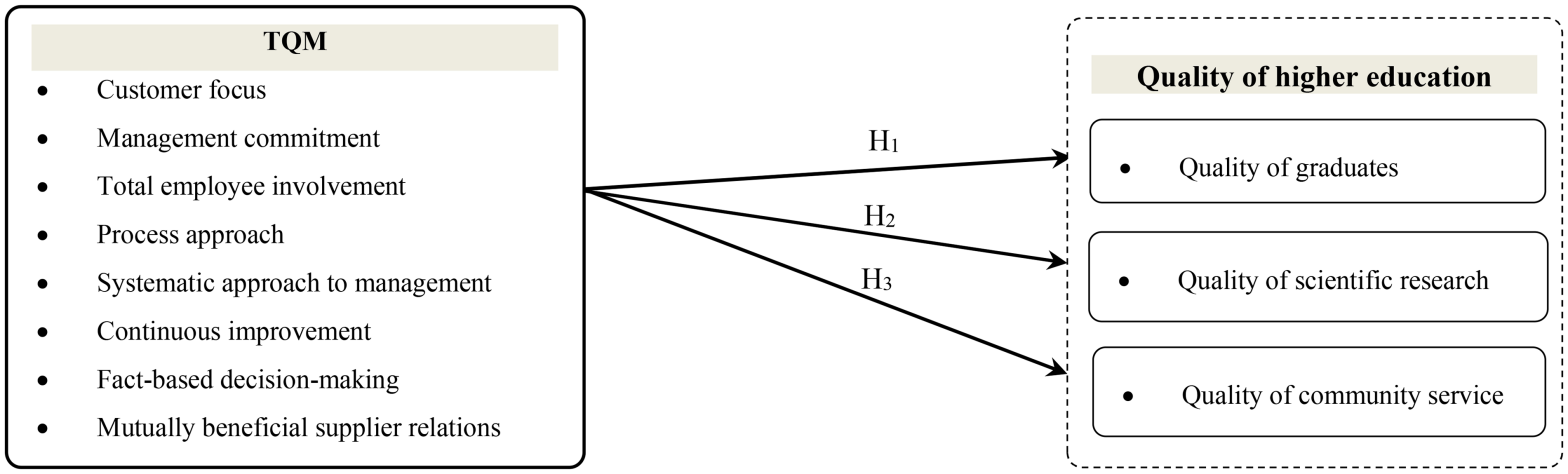
Research framework.

According to Browne and Cudeck (1992), the path model’s fit indices have been achieved; therefore, the relative Chi-square value is less than 5 (3.373), signifying that the study’s suggested model is consistent with the real data. The comparative fit index (1.000) and the Tucker Lewis index (0.997) are all very close to one, indicating that the study’s hypothetical model is far from zero (which assumes no association between the study variables), as well as a value of RMSEA less than 0.08 (0.062). All of this leads us to accept both the Research framework (Figure 2) and the hypotheses provided in Table 8.

**Table 8:** Direct effects in the path analysis model.

Effect type	Path	Estimate	S.E.	C.R.	*p*-Value	Hypothesis	Results
Direct	TQM → Quality of graduates	0.989	0.017	58.174	***	H_1_	Supported at 0.01
	TQM → Quality of community service	0.989	0.017	58.174	***	H_2_	Supported at 0.01
	TQM → Quality of scientific research	0.974	0.007	133.384	***	H_3_	Supported at 0.01

***Supported at 0.01.

Table 8 shows that total quality management has a significant, positive, and large impact on improving the quality of higher education. At the first level, total quality management has a 98.9% impact on improving the quality of graduates and improving the quality of community service, and at the second level, total quality management has a 97.4% impact on improving the quality of scientific research. At the level of significance *p* = 0.01, this leads us to reject the null hypotheses and accept the alternative hypotheses (H1, H2, and H3).

#### Qualitative results

**Figure 3:**
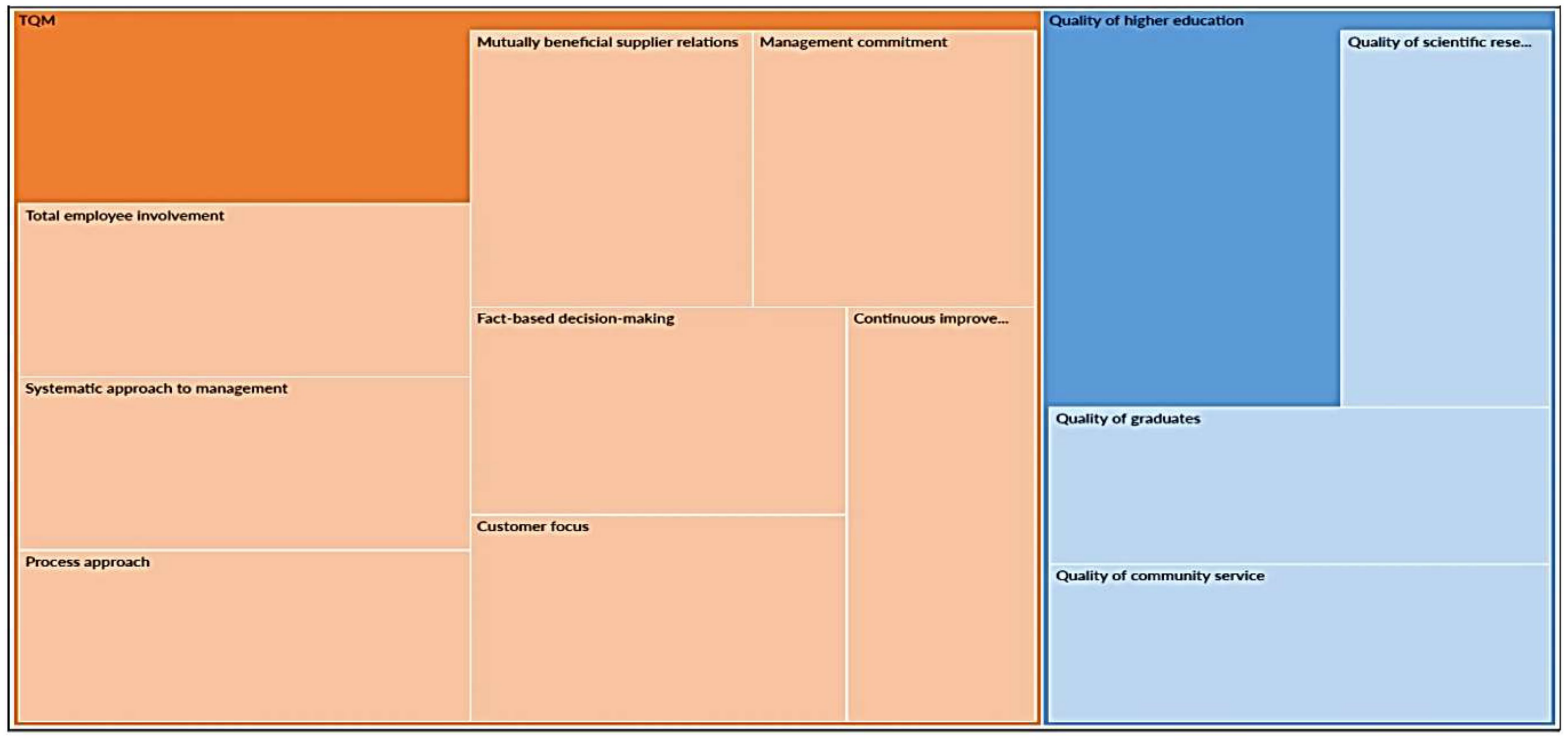
Matrix query of thematic analysis.

According to Clarke and Braun (2018), thematic analysis is not just a way of describing and reducing data; it is also a way of interpreting, describing, and summarizing the levels of discourse (codes or study variables) to check the validity of hypotheses. The following tendencies emerged from respondents’ assessments on Algerian universities’ TQM experience, giving to NVivo12 outputs:

From the thematic analysis (Figure 3), several implications can be reached. First, the respondents’ discussions were equally focused on the variables and dimensions of TQM, as they believe they are all crucially significant. Second, the interviewees’ discourse was similarly focused on the variables and dimensions of higher education quality, since they believe they are all equally important. Finally, there is a gap in the respondents’ discourse because their focus was on TQM rather than the quality of higher education, that is, because the implementation of TQM principles is the base for obtaining quality in Algerian higher education supported by Khelifa et al. (2013) and Keffane et al. (2020).

**Figure 4:**
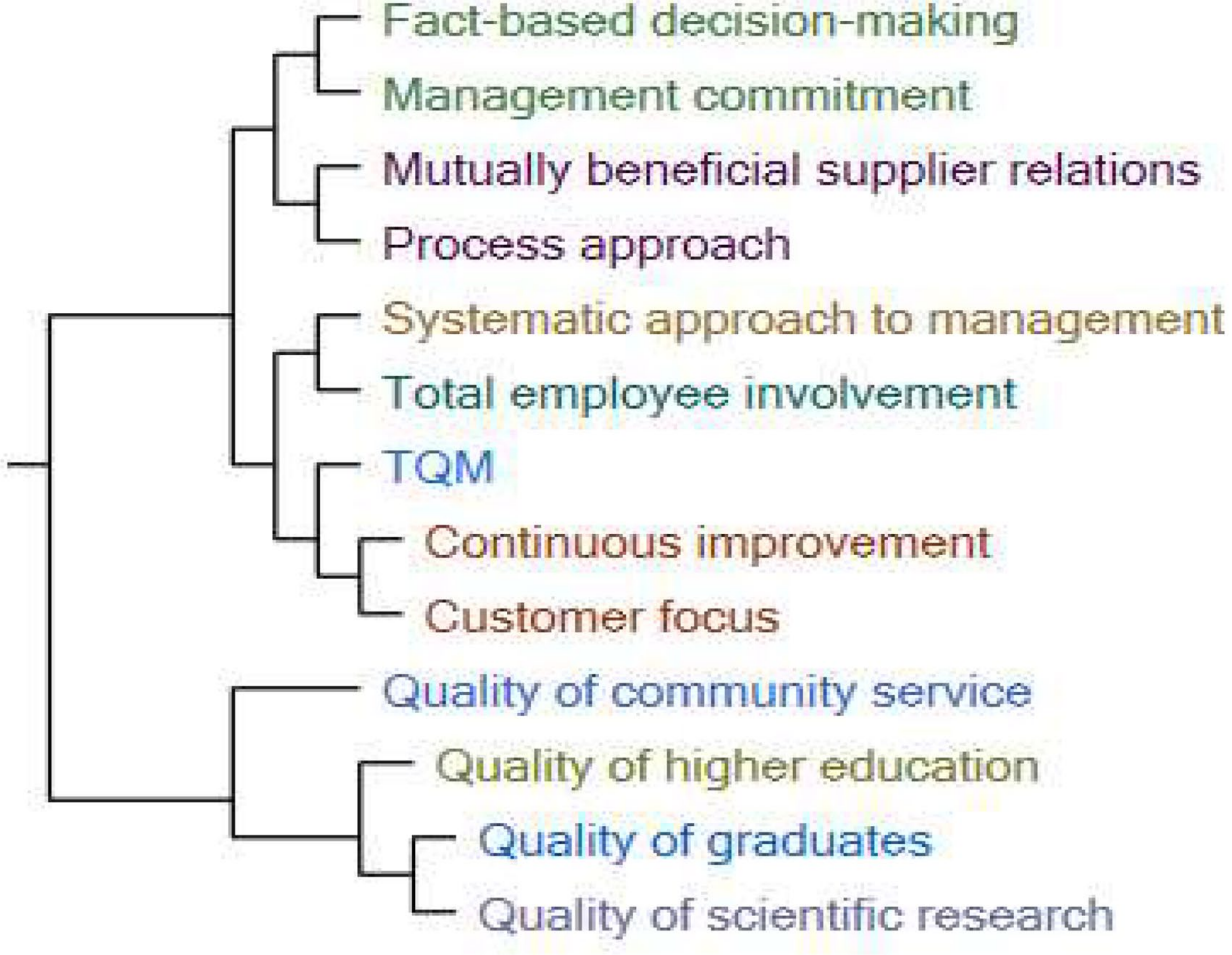
Cluster analysis of study variables.

Cognitive mapping, according to Eden, Jones, and Sims, is a modeling technique that tries to depict ideas, beliefs, values, and attitudes, as well as their links to one another, in a form that can be studied and analyzed (Northcott, 1996). According to this approach, Figure 4 shows the relationship between the study variables based on the cluster analysis results.

Figure 4 of the cluster analysis shows that there are three levels of relationships between the study variables: first, the relationship of total quality management with its dimensions, second, the relationship of higher education quality with its dimensions, and finally, the relationship of total quality management with higher education quality. The following table, based on Pearson’s coefficient, shows the relationship between independent and dependent variables in the study model.

Table 9 shows that there is a moderately positive relationship between all dimensions of TQM and all dimensions of higher education quality, with all Pearson’s correlation coefficients limited between 0.33 and 0.66. This is in line with the findings of quantitative analysis and path analysis, which all demonstrated the hypothesis’ validity (H1, H2, and H3).

**Table 9:** Pearson’s correlation coefficient.

Code A	Code B	Pearson’s correlation coefficient
Continuous improvement	Quality of graduates	0.399305
Customer focus	Quality of graduates	0.399305
Fact-based decision-making	Quality of graduates	0.399305
Management commitment	Quality of graduates	0.399305
Mutually beneficial supplier relations	Quality of graduates	0.399305
Process approach	Quality of graduates	0.399305
A systematic approach to management	Quality of graduates	0.399305
Total employee involvement	Quality of graduates	0.399305
TQM	Quality of graduates	0.399305
Continuous improvement	Quality of scientific research	0.399305
Customer focus	Quality of scientific research	0.399305
Fact-based decision-making	Quality of scientific research	0.399305
Management commitment	Quality of scientific research	0.399305
Mutually beneficial supplier relations	Quality of scientific research	0.399305
Process approach	Quality of scientific research	0.399305
A systematic approach to management	Quality of scientific research	0.399305
Total employee involvement	Quality of scientific research	0.399305
TQM	Quality of scientific research	0.399305
Continuous improvement	Quality of community service	0.390094
Customer focus	Quality of community service	0.390094
Fact-based decision-making	Quality of community service	0.390094
Management commitment	Quality of community service	0.390094
Mutually beneficial supplier relations	Quality of community service	0.390094
Process approach	Quality of community service	0.390094
A systematic approach to management	Quality of community service	0.390094
Total employee involvement	Quality of community service	0.390094
TQM	Quality of community service	0.390094
Continuous improvement	Quality of higher education	0.337994
Customer focus	Quality of higher education	0.337994
Fact-based decision-making	Quality of higher education	0.337994
Management commitment	Quality of higher education	0.337994
Mutually beneficial supplier relations	Quality of higher education	0.337994
Process approach	Quality of higher education	0.337994
A systematic approach to management	Quality of higher education	0.337994
Total employee involvement	Quality of higher education	0.337994
TQM	Quality of higher education	0.337994

## Discussion and implications

This paper aims to look at the impact of TQM on higher education quality from three perspectives (quality of graduates, quality of scientific research, and quality of community service). The study employed a mixed approach, with quantitative data from 610 questionnaires distributed to Algerian university students being analyzed *via* path analysis, and qualitative data from a structured interview with 24 professors who are members of the quality cells being analyzed *via* NVivo.

Our method is unique in that we used mixed methods to investigate the impact of TQM on three levels (quality of graduates, quality of scientific research, and quality of community service). There is a direct and significant impact of TQM on the quality of graduates in Algerian universities, a direct and significant impact of TQM on the quality of scientific research in Algerian universities, and a direct and significant impact of TQM on the quality of community service in Algerian universities, among other findings. The qualitative and quantitative results were both in agreement.

First, regarding the impact of TQM on the quality of graduates, which was found to be significant at 0.01 with a path coefficient estimated at 0.989, it was found that the qualitative correlation coefficient was 0.399305. These results are broadly consistent with several previous studies (Willis and Taylor, 1999; Sakthivel et al., 2005; Lin et al., 2012; Dahil and Karabulut, 2013; Martínez-Gómez et al., 2018, 2020). While it differs from some studies that acknowledge that the effect is weak (Koch, 2003). Second, the effect of TQM on the quality of scientific research was determined to be significant at 0.01 with a path coefficient of 0.974, and the qualitative correlation coefficient was found to be 0.399305. These findings are in line with those of several earlier research (Anninos, 2007; Jusoh et al., 2008; Mashagba, 2014; Zwain et al., 2017; Lanati, 2019). These results differ from some studies, Koch (2003) emphasized the reasons for the weak impact of TQM in higher education. Third, with a path coefficient of 0.0.989 and a qualitative correlation coefficient of 0.390094, the influence of TQM on the quality of community service was assessed to be significant at 0.01. These findings are consistent with those of previous studies (Nogueiro et al., 2011, 2017, 2022; Khoja et al., 2017; Banerjea, 2018; Yeung, 2018; Castillo, 2020), while these results differ from Koch (2003) which confirmed that the effect is small and identified the reasons for this.

Finally, all these results confirm the existence of a significant role of total quality management in improving the quality of higher education in Algerian universities, both quantitatively and qualitatively. This has been discussed and confirmed in several similar studies (Khelifa et al., 2013; Mashagba, 2014; Zwain et al., 2014, 2017; Barka and Ilhem, 2016; Psomas and Antony, 2017; Khan et al., 2019; Keffane et al., 2020; Belimane and Chahed, 2021), while Koch (2003) study remains, which always confirms that this effect is weak.

Accordingly, the study gave an addition compared to previous studies, especially those that were completed in Algerian universities. It demonstrated the existence of direct and significant effects of total quality management in improving the quality of graduates, the quality of scientific research, and the quality of community service, using quantitative methods (path analysis) and qualitative methods (content analysis). This is what the directors of Algerian higher education institutions are looking for, that is, the importance, advantages, and effects of the application of total quality management before looking for how to apply it, those indicated by Khelifa et al. (2013) Barka and Ilhem (2016), Keffane et al. (2020), and Belimane and Chahed (2021).

At least four limitations apply to the conclusions of this study. First, the study did not assess the influence of TQM on higher education quality, comparing universities, academic environments, and students, as well as the variations resulting from changes in professors’ perspectives. This may necessitate a meta-study that compiles the findings of various research conducted in various settings. Second, one outstanding question is whether the quality of higher education is influenced by other variables such as student and faculty levels, curricula and programs, the administrative system, and so on. This may require another empirical investigation. Third, the most significant limitation is that, as a result of the Corona epidemic, the quality of higher education has altered, with universities becoming increasingly reliant on e-learning, and we must discuss the quality and value of this system. Finally, we may need data from a bigger sample and may need to utilize other statistical approaches, such as analysis of variance, to quantify the impact between the research variables. Future research trends are being evaluated as a result of these limitations.

## Conclusion

The main objective of this study was to investigate the impact of TQM on higher education quality in Algerian universities by examining three sub-problems (impact of TQM on quality of graduates, impact of TQM on quality of scientific research, and impact of TQM on quality of community service) using a quantitative analysis of 610 questionnaires distributed to Algerian university students and qualitative analysis of 24 structured interviews with professors’ members of the quality cells.

To summarize the quantitative findings, the correlation matrix shows that TQM and its dimensions (continuous improvement, customer focus, fact-based decision-making, management commitment, mutually beneficial supplier relationships, process approach, and systematic approach to management) have a positive significant correlation with higher quality. TQM has a direct and considerable impact on the quality of graduates, scientific research, and community service at Algerian universities, according to the SEM results or path analysis model.

The qualitative findings reveal three levels of relationships between the study variables: first, the relationship between total quality management and its dimensions, second, the relationship between higher education quality and its dimensions, and third, the relationship between total quality management and higher education quality.

The study’s findings can benefit university administrators, leaders, and policymakers in the Ministry of Higher Education and Scientific Research on several levels. The first is to go from the stage of self-assessment to the stage of establishing a quality management system. Second, achieving institutional and programmatic academic accreditation is a priority. Third, a comprehensive quality management approach for use in Algerian universities is being developed. Fourth, working on creating policies to help universities advance in their international classification. Fifth, policy-makers and directors of higher education institutions in Algeria must work on preparing clear strategies to adopt and apply the principles of total quality management and establish a quality management system to improve the quality of higher education (improving the quality of graduates, improving the quality of scientific research, and improving the quality of community service) by relying on an integrated quality system in which all stakeholders participate (professors, administrators, students, workers, and external community), which allows the classification of Algerian universities in the first ranks in the international classifications of universities.

Work on the remaining challenges is continuing and will be published in future studies, based on the positive discoveries presented in this paper. The next stage of our research will be a meta-analysis of the impact of TQM on higher education quality. Other issues to be addressed include the disparities in impacts between universities, the impact of other variables such as student and faculty levels, curricula and programs, and the administrative system on higher education quality.

## Data availability statement

The original contributions presented in the study are included in the article/supplementary material, further inquiries can be directed to the corresponding author.

## Author contributions

Study design, data collection and analysis, and manuscript editing and writing were all conducted by FY, KC and SB. All authors contributed to the article and approved the submitted version.

## Funding

The authors extend their appreciation to the General Directorate of Scientific Research and Technological Development (GDSRTD), Ministry of Higher Education and Scientific Research, Algeria, to fund this work.

## Conflict of interest

The authors declare that the research was conducted in the absence of any commercial or financial relationships that could be construed as a potential conflict of interest.

## Publisher’s note

All claims expressed in this article are solely those of the authors and do not necessarily represent those of their affiliated organizations, or those of the publisher, the editors and the reviewers. Any product that may be evaluated in this article, or claim that may be made by its manufacturer, is not guaranteed or endorsed by the publisher.
